# Humoral Responses and Ex Vivo IFN-γ Production after Canine Whole Blood Stimulation with *Leishmania infantum* Antigen or KMP11 Recombinant Protein

**DOI:** 10.3390/vetsci9030116

**Published:** 2022-03-04

**Authors:** Pamela Martínez-Orellana, Noemí González, Antonella Baldassarre, Alejandra Álvarez-Fernández, Laura Ordeix, Paola Paradies, Manuel Soto, Laia Solano-Gallego

**Affiliations:** 1 Departament de Medicina i Cirurgia Animal, Facultat de Veterinària, Universitat Autònoma de Barcelona, 08193 Bellaterra, Spain; pamela.martinez.phd@gmail.com (P.M.-O.); noglezmartinez@gmail.com (N.G.); alejalfe@gmail.com (A.Á.-F.); laura.ordeix@uab.cat (L.O.); 2 Dipartimento delle Emergenze e dei Trapianti d’ Organo, Sezione Cliniche Veterinarie, Università degli Studi di Bari Aldo Moro, 70124 Bari, Italy; a.baldassarre12@studenti.uniba.it (A.B.); paola.paradies@uniba.it (P.P.); 3 Departamento Biología Molecular, Centro Biología Molecular Severo Ochoa, Universidad Autónoma de Madrid, 28049 Madrid, Spain; msoto@cbm.csic.es

**Keywords:** leishmaniosis, serology, dog, cell-mediated immunity, diagnosis

## Abstract

The effect of *Leishmania infantum* soluble antigen (LSA) and recombinant Kinetoplastid Membrane Protein 11 (rKMP11) on the induction of ex vivo specific IFN-γ (*n* = 69) and antibody responses (*n* = 108) was determined in dogs. All dogs were tested for serological response to both antigens and divided into Group 1: healthy (Asturias, Spain, *n* = 26), Group 2: sick (*n* = 46), Group 3: healthy Ibizan hounds (Mallorca, Spain, *n* = 22) and Group 4: healthy (Bari, Italy, *n* = 14). Antibody levels were higher for LSA when compared to rKMP11 (*p* = 0.001). Ibizan hounds were all seronegative to rKMP11 and 18% were low seropositive to LSA. Sick dogs presented higher antibody response to both antigens compared to the rest of the groups (*p* < 0.0001). All groups showed higher IFN-γ levels after LSA compared to rKMP11 responses (*p* < 0.05). The highest response to LSA was found in Ibizan hounds (*p* < 0.05). IFN-γ to LSA and rKMP11 stimulation was observed in 34% and in 2.8% of the sick dogs, respectively. Here, we demonstrated that anti-rKMP11 antibodies are mainly present in dogs with moderate to severe disease. Furthermore, cellular immune response measured by specific ex vivo IFN-γ production was more intense to LSA than stimulated to rKMP11.

## 1. Introduction

Canine leishmaniosis (CanL) is a zoonotic vector-borne disease transmitted by phlebotomine sandflies. *Leishmania infantum* is the species responsible for the clinical form of this disease in Europe where the domestic dog is the principal reservoir host [1,2]. Canine leishmaniosis exhibits a wide distribution in the Mediterranean basin [3], Middle East [4] and South America [5].

Canine *L. infantum* infection is a potentially severe and fatal disease [6], that displays a broad range of clinical manifestations and immune responses which are characterized by different clinical outcomes, prognosis and treatment options [7]. Guidelines that establish a clinical staging system are currently applied [8]. The outcome of the infection is highly variable depending on each dog’s immune response among other factors [7]. However, two extremes of this clinical spectrum are represented by: (1) healthy dogs that develop a T cell immune response, characterized by a protective production of proinflammatory cytokines which leads to infection control, and (2) sick dogs characterized by a marked humoral immune response and lack or diminished cell-mediated immunity and parasite dissemination [7,9].

IFN-γ has been studied in several organs during CanL independently of the clinical staging and different states of infection [10]. Thus, there are limited studies regarding sick dogs with different degrees of disease severity [11,12]. Studies performed confirm that sick dogs with the absence of production of *L. infantum* specific IFN-γ after stimulation of whole blood elicit a strong humoral response and have moderate to very severe disease, demonstrating that IFN-γ plays a key role in forecasting the prognosis of the disease [13].

Several surface proteins of the *Leishmania* parasites have been studied for their immunogenic properties. Kinetoplastid membrane protein 11 (KMP11) is a conserved protein, widely distributed among trypanosomatids [14] but poorly studied in CanL. The biological function of the *L. infantum* KMP11 protein remains unknown. However, some studies have suggested a role on parasite mobility of *L. infantum* [15], parasite survival within the phagolysosome of the host cell when infected with *L. amazonensis* [16], as well as stage and growth-phase developmental control in *L. infantum* [17]. During *Leishmania* infection, KMP11 links to one of the major parasite surface molecules, lipophosphoglycan (LPG), leading to the stimulation of the immune response [18,19].

Therefore, characterization of the immunogenic and protective capacity in the dog in response to different antigens such as KMP11 and LSA is needed for the development of standardized assays to estimate T-cell mediated and humoral immune responses as well as immunotherapeutic tools [20]. The purpose of this study was to investigate the antibody responses induced by LSA and recombinant kinetoplastid membrane protein 11 (rKMP11) in serum samples from healthy and sick dogs by in house enzyme-linked immunosorbent assay (ELISA) and the effects of LSA and rKMP11 antigens on the ex vivo induction of specific IFN-γ cytokine responses in whole blood from dogs with different states of infection.

## 2. Materials and Methods

### 2.1. Dogs

A total of 108 dogs were enrolled during October 2016 to November 2018. Both sexes were represented, with 57 females and 51 males. The median of age was four years, with a range from five months to 17 years. Eighty-three purebred dogs belonging to 28 breeds and 16 mixed breed dogs were included.

Dogs were classified into four groups according to their clinical status. Physical examination and serological diagnosis for *L. infantum* were carried out in all dogs studied as described below in Section 2.3. Group 1 included 26 control seronegative apparently healthy dogs. The dogs were from the Asturias region in northern Spain, a very low endemicity area of leishmaniosis [21,22]. The median of age was 52-months, with a range from 6- to 144-months-old, including 14 females and 12 males. Dog breeds included one Schnauzer, two Spanish mastiffs, one Afghan hound, one Fox Terrier, one German Shepherd, two Labrador Retrievers, two Spanish water dogs, five Border Collies, one Bobtail, one Bernese Mountain dog, one Bearded collie, one Berger De Brie and seven mixed breeds. 

Group 2 included sick dogs with a mild to severe clinical stage of leishmaniosis (*n* = 46) [23]. The median of age was 47.7 months with a range from 5- to 204-months. There were 25 females and 21 males. The following breeds were represented: two Yorkshire Terriers, four American Staffordshire terriers, three Dachshund Teckels, one Australian shepherd, two Bulldogs, seven Labrador Retrievers, one Andalusian wine-cellar, one Breton, one American pit bull terrier, one English setter, four Boxers, one Pinscher, two German Shepherds, one Ibizan hound, one Doberman, one Rottweiler, one French Bulldog, one Dalmatian, one Beagle, one Schnauzer, one Greyhound, one German Braco, one Akita Inu and five mixed breeds. All dogs were subjected to a routine wellness examination, a full blood count, a biochemical profile including creatinine, urea, total proteins, alanine transaminase and total cholesterol, protein serum electrophoresis, urinalysis with urinary protein/creatinine ratio and quantitative serology for the detection of *L. infantum* specific antibodies by means of a serial dilution in house ELISA [24,25]. Cytological evaluation of any lesion or cutaneous histology and/or immunohistochemistry for *Leishmania* were also performed when needed as described elsewhere [26]. Sick dogs were classified in four clinical stages (stage I-mild disease, II-moderate disease, III-severe disease and IV-very severe disease) at the time of diagnosis, as previously described [8]. Twenty-nine dogs were classified in LeishVet stage II (moderate disease) with 22 dogs subdivided into stage IIa and seven dogs into stage IIb. The remaining dogs were categorized in stage I (*n* = 5), stage III (*n* = 8) and stage IV (*n* = 4). All dogs in stage I presented *L. infantum-*associated papular dermatitis. The rest of the dogs presented typical clinicopathological findings and moderate to high antibody levels.

Group 3 included 22 apparently healthy Ibizan hound dogs from the island of Mallorca, a highly endemic area of leishmaniosis in Spain. They were 14 females and 8 males with a mean age of 31.4 months and an age range of 7- to 72-months. 

Finally, Group 4 included 14 apparently healthy dogs from the city of Bari, a highly endemic area of leishmaniosis in Italy. There were nine females and five males with a mean of age of 63.4 months with a range from 12- to 120-months; most were mixed breeds (*n* = 13) and there was one English Setter.

### 2.2. Antigen Preparation

*Leishmania* soluble antigen was prepared from stationary phase promastigotes of *L. infantum* (MHOM/MON-1/LEM 75), as previously described [27]. 

The KMP11 coding region (LinJ.35.2260) was cloned in the vector pET-28b for expression in *Escherichia coli* BL21 (DE3), as previously described [28,29]. Briefly, the gene was amplified by PCR using genomic DNA from the *L. infantum* JPC clone (MCAN/ES/98/LLM-724) and the next specific primers: Forward, 5′-CCATGGCCACCACGTACGAGG-3′ and Reverse, 5′-GGATCCTTACTTGGACGGGTACTGCG-3′. For subcloning purposes, the insert was obtained by digestion with NcoI/BamHI and cloned into the corresponding sites of the pET28a (+) *E. coli* expression vector (Sigma-Aldrich, Burlington, VT, USA). Then, *E. coli* (BL21 strain; Sigma-Aldrich, Burlington, VT, USA) transfected with the recombinant plasmids were employed for over-expression of the KMP11 proteins. Next, the solubilized KMP-11 was recovered from the supernatant and the purification was performed by an ammonium sulfate fractionated precipitation protocol. Protein concentration was estimated by the Bradford method using the Bio-Rad Protein assay (Bio-Rad, Hercules, CA, USA) according to manufacturer’s instructions and the purity was determined by SDS-PAGE and Coomassie staining as previously described [30], and was good. 

### 2.3. Serological Diagnosis by ELISA 

#### 2.3.1. *Leishmania infantum* ELISA

*Leishmania infantum*-specific antibodies were measured by endpoint ELISA in all dogs studied, as previously described [24]. Briefly, samples were diluted to 1:800 and incubated in *L. infantum* antigen-coated plates (20 μg/mL). Next, plates were incubated with Protein A (Thermo Fisher Scientific, Whaltham, MA, USA, dilution 1:30,000) and developed by adding the substrate solution o-phenylenediamine and substrate buffer (SIGMAFAST OPD, Sigma-Aldrich, Burlington, VT, USA). The reaction was stopped with 50 μL of 2.5 M H_2_SO_4_. Absorbance values were read at 492 nm by an automatic reader (ELISA Reader Anthos 2020). Samples were run in duplicate and all samples with an optical density (OD) equal to or higher than three were studied using a two-fold serial dilution that started at 1:800 and continued for 9 to 11 further dilutions. Positive and negative sera were included in all plates as controls. The result was quantified as ELISA units (EU) relative to a positive canine calibrator serum set at 100 EU. The cut-off was established at 35 U, as previously described.

#### 2.3.2. KMP11 ELISA

All dogs were also tested for rKMP11-specific antibodies following the ELISA protocol as previously described for *L. infantum*-specific antibodies [25] with slight variations. Briefly, the sera samples were diluted to 1:800. rKMP11 antigen-coated plates (1 μg/mL) in carbonate-bicarbonate buffer were incubated over night at 4 °C. The concentration of rKMP11 antigen-coated plates of 1 μg/mL was established based on a standard curve of antigen (data not shown). Thereafter, the plates were incubated with Protein A conjugated to horseradish peroxidase (Thermo Fischer Scientific, Waltham, MA, USA, dilution 1:40,000) for 1 h at 37 °C. Samples were run in duplicate, positive and negative sera were included in all plates as controls. The result was quantified as ELISA units (EU) related to a positive canine serum set at 100 EU. The cut-off was established at 24 EU (mean + 4 SD of values from 67 dogs from non-endemic area).

### 2.4. Whole Blood Assay

A total of 69 dogs were conveniently selected for determination of ex vivo IFN-γ specific concentrations after *L. infantum* or rKMP11 stimulation of whole blood (Group 1; *n* = 25, Group 2; *n* = 15, Group 3; *n* = 16 and Group 4, *n* = 13) as previously described [13,31]. Briefly, five hundred μL of heparinized blood was diluted to a ratio of 1:10 with RPMI 1640 complete medium (Biowest, Nuaillé, Frances) per each well and incubated in 12-well flat bottom plastic culture Costar^®^ plates 3596 (Corning, Corning, NY, USA). Experimental treatments were as follows: (1) medium alone, (2) LSA at 10 μg/mL, (3) recombinant kinetoplastic protein 11 (rKMP11) at 10 μg/mL and (4) mitogen ConA (100 mg, Medicago, Uppsala, Sweden) at of 10 μg/mL. All treatment conditions were incubated for 5 days at 37 °C in 5% of CO_2_ air. Then, blood was collected and centrifuged at 300× *g* for 10 min and the supernatant was collected and stored at −80 °C until used. 

### 2.5. Sandwich ELISA for the Determination of IFN-γ 

Cytokine analysis of IFN-γ (*n* = 69) was performed according to the manufacturer’s instructions (DuoSet^®^ ELISA by -R&D Systems, Minneapolis, MN, USA) using a 96 well cell Costar^®^ plate flat bottom (Corning, Corning, NY, USA). Slight modifications were done for IFN-γ ELISA. The standard curve for IFN-γ started with 2000 pg/mL and two-fold dilutions were made until reaching a 7.8 pg/mL concentration. Dogs were classified as IFN-γ producers when *L. infantum* or rKMP11 specific IFN-γ concentrations were ≥100 pg/mL after subtracting the medium alone. 

### 2.6. Statistical Analysis

A non-parametric Mann–Whitney U test was used to compare the groups. A non-parametric Wilcoxon signed-rank test was used to compare paired continuous variables. Differences were considered significant with a 5% significance level (*p* < 0.05). The statistical analysis was performed using SPSS 22.0 for Windows software (IBM, Armonk, NY, USA).

## 3. Results

### 3.1. Antibody Response 

**Figure 1:**
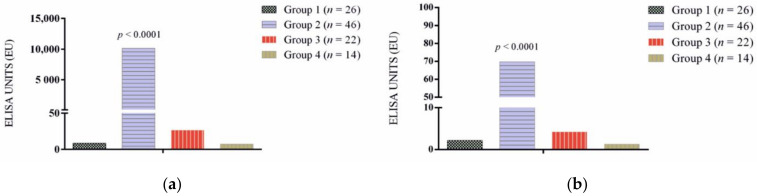
Specific (**a**) *L infantum* and (**b**) rKMP11 antibodies levels in apparently healthy non-infected dogs from a low endemicity area, Asturias, Spain (Group 1), sick dogs from Catalonia, Spain (Group 2) apparently healthy Ibizan hounds from a high endemicity area, Mallorca, Spain (Group 3) and apparently healthy dogs from a high endemicity area, Bari, Italy (Group 4).

**Figure 2:**
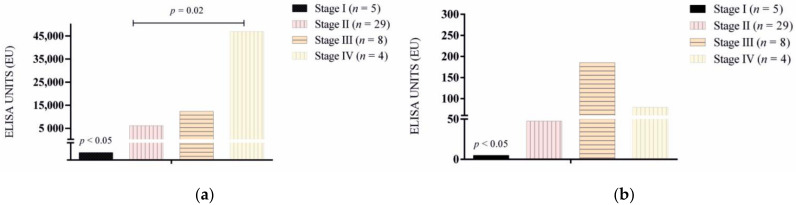
Specific (**a**) *L. infantum* and (**b**) rKMP11 antibody levels in sick dogs (Group 2) classified based on LeishVet clinical staging.

The results of antibody levels for *L. infantum* and rKMP11 are shown in Figure 1a,b and Figure 2a,b. In general, antibody levels were higher for *L. infantum* when compared to the rKMP11 antigen (*p* = 0.001). Seropositive responses by groups to *L. infantum* and rKMP11 antigens are summarized in Table 1. 

All healthy dogs from Asturias (Group 1) and Bari (Group 4) were seronegative with both antigens. Ibizan hound resistant dogs (Group 3) were all seronegative to rKMP11 and 18% had low seroreactivity to the *L. infantum* antigen. Catalonian sick dogs (Group 2) presented a higher antibody response to both antigens compared to the rest of the groups (*p* < 0.0001) (Figure 1a,b). The results of antibody levels based on clinical staging showed that stage I sick dogs presented lower antibodies when compared with the rest of the stages for both antigens (*p* < 0.05) (Figure 2a,b). No significant differences were found between LeishVet stages II, III and IV for rKMP11 (Figure 2b), while higher *L. infantum* antibodies (Figure 2a) were found in stage IV when compared to stage II (*p* = 0.02). 

### 3.2. Ex Vivo IFN-γ-Release Whole Blood Assay

**Figure 3:**
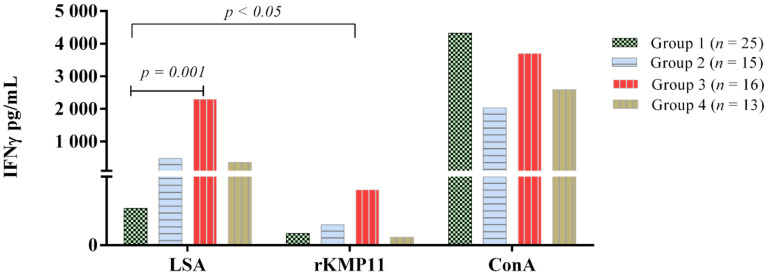
Specific ex vivo IFN-γ concentration after *L. infantum*, rKMP11 and ConA stimulation in apparently healthy non-infected dogs from a low endemicity area, Asturias, Spain (Group 1), sick dogs from Catalonia, Spain (Group 2), apparently healthy Ibizan hounds from a high endemicity area, Mallorca, Spain (Group 3) and apparently healthy dogs from a high endemicity area, Bari, Italy (Group 4).

The results of ConA, rKMP11 and *L. infantum* specific IFN-γ concentrations of the groups studied and based on clinical staging and IFN-γ classification are displayed in Figure 3.

The mean concentration of IFN-γ after antigen stimulation was compared, and most groups (*n* = 69) showed significantly higher concentrations of IFN-γ after LSA stimulation when compared to rKMP11 (Group 1; *p* = 0.09, Group 2; *p* = 0.043, Group 3; *p* = 0.002, Group 4, *p* = 0.001). Group 1 showed a reduced response, almost lacking a response to LSA, which was statistically significantly lower when compared to Group 3 (*p* = 0.001) (Figure 3). Conversely, the highest IFN-γ concentration to LSA and rKMP11 were found in Group 3 (*p* < 0.05). No statistically significant differences within groups were found when specific IFN-γ concentration to rKMP11 was compared.

The specific IFN-γ response by groups to the *L. infantum* and rKMP11 antigens are summarized in Table 2. In general, twenty-four out of a total 69 dogs (34%) were classified as IFN-γ producers after LSA stimulation and two (2.8%) after rKMP11 stimulation. 

## 4. Discussion

The study presented here demonstrated that sick and healthy dogs presented differences in serological response and specific ex vivo IFN-γ concentrations when blood was stimulated with *L. infantum* or rKMP11 antigens. 

First, we analyzed and compared the serological response of all dogs (*n* = 108) to both antigens. Antibody levels and frequencies of seropositive dogs were significantly higher to LSA when compared to the rKMP11 antigen. In agreement with the data presented here, a study of experimental *L. infantum* infection that compared asymptomatic and oligosymptomatic beagle dogs showed a lower antibody response to KMP11 when compared to LSA [20]. Furthermore, a study performed in Brazil with serum samples from clinically ill and healthy *Leishmania* infected dogs also showed lower antibody response to KMP11 when compared to LSA [32]. However, it is important to highlight that KMP-11 is present in a wide range of trypanosomatids, which are common pathogens in South America but not in Europe, that can induce cross-reactivity in the sera of animals from those areas [33,34]. Supporting this statement, a previous study that compared the amino acid sequence of KMP11 protein of *Trypanosoma cruzi* revealed 86% identity with the KMP11 from various *Leishmania* species [35]. In cases in which identification and discrimination of the etiological agent are needed, further isolation and sequence of the pathogen must be necessary. 

In agreement with previous studies, dogs with mild disease (stage I) presented the lowest antibody levels in response to both antigens [13,31]. As expected, the strongest humoral response was found in dogs with moderate to severe clinical stages, which is in line with the preceding data [24]. Dogs with clinical leishmaniosis presented a diminished *L. infantum* specific T-cell mediated immunity [36] which is clearly observed in moderate to severe cases of CanL [31]. *Leishmania*-specific IFN-γ production in stimulated blood of dogs [37,38], as well as in mice and humans has been associated with a protective phenotype [7]. However, dogs with mild to moderate illness might frequently present T-cell mediated immunity [39,40], which is in agreement with the present results.

Interestingly, a high proportion of dogs with clinical leishmaniosis presented a strong *L. infantum* specific ex vivo IFN-γ concentration in contrast with only two dogs after rKMP11 stimulation. Little is known about specific ex vivo IFN-γ response after whole blood rKMP11 stimulation. However, the immunogenic properties of KMP11 have been studied in dogs and hamsters [41]. One study of experimental *L. donovani*-infection in hamsters induced a mixed Th1/Th2 T cellular immune response, with high levels of IFN-γ, TNF-α, IL-4 and IL-12 but a lack of IL-10. Also, the study performed on a hamster model of infection demonstrated that KMP11 was able to protect animals against disease development [41]. Another study revealed that the stimulation of PBMCs from *L. infantum*-infected dogs with LSA induced a strong proliferation and IFN-γ gene expression and KMP11 induced only a moderate increase of IFN-γ gene expression [20]. Previous studies in human patients with cutaneous leishmaniasis (CL) and mucosal leishmaniasis (ML) due to *Leishmania braziliensis* demonstrated that KMP11 was able to downregulate IFN-γ production [42]. Moreover, KMP11 efficiently induced more secretion of IL-10 than IFN-γ. In agreement with the data presented here, ML patients presented higher IFN-γ production in response to *Leishmania* antigens compared to KMP11 when PBMC were stimulated with the different recombinant antigens [42].

It is also important to note that sick IFN-γ producing dogs were classified in the lower clinical stages, such as stage I, stage IIa and stage IIb in the case of *L. infantum* stimulation, and stage I in the case of rKMP11 stimulation. Moreover, dogs with the highest antibody response also presented the lowest LSA or rKMP11 IFN-γ production. As we demonstrated previously, differences in *L. infantum-*specific cytokine profiles in canine stimulated blood were found from several clinical stages of leishmaniosis [13,31]. 

Previous studies have also demonstrated that reduced cell-mediated immunity in clinical CanL revealed as the inability to respond to *Leishmania* antigen is correlated with the progression of infection [11,12,13,24]. This study supports the use of the ex vivo IFN-γ release whole blood assay to be included in the diagnosis and prognosis of dogs with moderate to severe clinical leishmaniosis together with serological testing. The IFN-γ response to both antigens together is lacking in the most clinically affected dogs accompanied by a high antibody response. *Leishmania infantum* specific T-cell mediated immunity responses in dogs with clinical leishmaniosis at different clinical stages have been evaluated [13]; however, here we presented the response to KMP11 antigen in dogs with different clinical stages for the first time. As demonstrated in several studies of human visceral leishmaniasis [43], assays that allow for the evaluation of the cellular immune response in dogs from endemic areas are an important clinical tool. 

The present results improve the knowledge on humoral and adaptive immunological responses of dogs in different areas of CanL endemicity. 

## 5. Conclusions

We demonstrated that the ex vivo ability of LSA in stimulating a cellular immune response measured by specific production of IFN-γ in the whole blood of dogs was more intense than the response exhibited to rKMP11.

Anti-KMP11 antibodies are mainly present in *L. infantum* in naturally infected dogs with moderate to severe disease. However, the limited detection of rKMP11 antibodies compared to *L. infantum* preclude the use of rKMP11 ELISA as a reliable diagnostic tool for *Leishmania* infection in canines.

## Figures and Tables

**Table 1:** Positive results to specific *L. infantum* and rKMP11 antibodies in apparently healthy non-infected dogs from Asturias (Spain), a low endemicity area (Group 1), sick dogs from Catalonia (Spain) (Group 2), apparently healthy Ibizan hound dogs from the high endemicity area of Mallorca (Spain) (Group 3), and apparently healthy dogs from the high endemicity area of Bari, Italy (Group 4).

Total Dogs (*n* = 108)	Positive Antibody Response	
	*L. infantum N* (%)	rKMP11 *N* (%)
Group 1 (*n* = 26)	0 (0)	0 (0)
Group 2 (*n* = 46)		
Stage I (*n* = 5) Stage IIa (*n* = 22) Stage IIb (*n* = 7) Stage III (*n* = 8) Stage IV (*n* = 4)	1 (3.1) 22 (47.8) 7 (15.2) 8 (17.3) 4 (8.6)	0 (0) 12 (26.0) 6 (13.0) 5 (10.8) 3 (6.5)
Group 3 (*n* = 22)	4 (18.1)	0 (0)
Group 4 (*n* = 14)	0 (0)	0 (0)
Total	46 (42.5)	26 (24)

*N* = number of dogs by group positive to the test, % = percentage of dogs by group positive to the test.

**Table 2:** Positive results to specific ex vivo IFN-γ response to *L. infantum* and rKMP11 in apparently healthy non-infected dog from Asturias, Spain, a low endemicity area (Group 1), sick dogs from Catalonia (Spain) (Group 2), apparently healthy Ibizan hound dogs from the high endemicity area of Mallorca (Spain) (Group 3) and apparently healthy dogs from the high endemicity area of Bari (Italy) (Group 4).

Total Dogs (*n* = 69)	IFNγ Response	
	LSA *N* (%)	rKMP11 *N* (%)
Group 1 (*n* = 25)	0 (0)	0 (0)
Group 2 (*n* = 15)		
Stage I (*n* = 6) Stage IIa (*n* = 6) Stage IIb (*n* = 2) Stage III (*n* = 1)	2 (13.3) 2 (13.3) 1 (6.6) 0 (0)	2 (13.3) 0 (0) 0 (0) 0 (0)
Group 3 (n = 16)	12 (75)	0 (0)
Group 4 (n = 13)	7 (53.8)	0 (0)
Total	24 (34.7)	2 (2.8)

*N* = number of dogs by group positive to the test, % = percentage of dogs by group positive to the test, LSA = *Leishmania* soluble antigen.

## Data Availability

The data presented in this study are available on request from the corresponding author.

## References

[1] Noli C., Saridomichelakis M.N. (2014). An update on the diagnosis and treatment of canine leishmaniosis caused by *Leishmania infantum* (syn. L. chagasi). Vet. J..

[2] Ready P.D. (2010). Leishmaniasis emergence in Europe. Eurosurveillance.

[3] Velez R., Ballart C., Domenech E., Abras A., Fernández-Arévalo A., Gómez S.A., Tebar S., Muñoz C., Cairó J., Gállego M. (2018). Seroprevalence of canine *Leishmania infantum* infection in the Mediterranean region and identification of risk factors: The example of North-Eastern and Pyrenean areas of Spain. Prev. Vet. Med..

[4] Tabbabi A. (2019). Review of Leishmaniasis in the Middle East and North Africa. Afr Health Sci..

[5] Dantas-Torres F. (2009). Canine leishmaniosis in South America. Parasit. Vectors..

[6] Pereira M.A., Santos R., Oliveira R., Costa L., Prata A., Gonçalves V., Roquette M., Vala H., Santos-Gomes G. (2020). Prognostic factors and life expectancy in canine leishmaniosis. Vet. Sci..

[7] Toepp A.J., Petersen C.A. (2020). The balancing act: Immunology of leishmaniosis. Res. Vet. Sci..

[8] Solano-Gallego L., Koutinas A.F., Miró G., Cardoso L., Pennisi M.G., Ferrer L., Bourdeau P., Oliva G., Baneth G. (2009). Directions for the diagnosis, clinical staging, treatment and prevention of canine leishmaniosis. Vet. Parasitol..

[9] Reis A.B., Giunchetti R.C., Carrillo E., Martins-Filho O.A., Moreno J. (2010). Immunity to *Leishmania* and the rational search for vaccines against canine leishmaniasis. Trends Parasitol..

[10] Rodríguez-Cortés A., Carrillo E., Martorell S., Todolí F. (2016). Compartmentalized immune response in leishmaniasis: Changing patterns throughout the disease. PLoS ONE.

[11] Strauss-Ayali D., Baneth G., Shor S., Okano F., Jaffe C.L. (2005). Interleukin-12 augments a Th1-type immune response manifested as lymphocyte proliferation and interferon gamma production in *Leishmania infantum*-infected dogs. Int. J. Parasitol..

[12] Boggiatto P.M., Ramer-Tait A.E., Metz K., Kramer E.E., Gibson-Corley K., Mullin K., Hostetter J.M., Gallup J.M., Jones D.E., Petersen C.A. (2010). Immunologic indicators of clinical progression during canine *Leishmania infantum* infection. Clin. Vaccine Immunol..

[13] Martínez-Orellana P., Marí-Martorell D., Montserrat-Sangrà S., Ordeix L., Baneth G., Solano-Gallego L. (2017). *Leishmania infantum*-specific IFN-γ production in stimulated blood from dogs with clinical leishmaniosis at diagnosis and during treatment. Vet. Parasitol..

[14] Stebeck C.E., Beecroft R.P., Singh B.N., Jardim A., Olafson R.W., Tuckey C., Prenevost K.D., Pearson T.W. (1995). Kinetoplastid membrane protein-11 (KMP-11) is differentially expressed during the life cycle of African trypanosomes and is found in a wide variety of kinetoplastid parasites. Mol. Biochem. Parasitol..

[15] Angel M., Jose F., Perez M., Soto M., Carlos M., Carlos L. (2001). Calcium-induced conformational changes in *Leishmania infantum* kinetoplastid membrane protein-11. J. Biol. Inorg. Chem..

[16] Matos D.C.S., Faccioli L.A.P., Cysne-Finkelstein L., Mello De Luca P., Corte-Real S., Armôa G.R.G., Blanco Lemes E.M., Decote-Ricardo D., Mendonça S.C.F. (2010). Kinetoplastid membrane protein-11 is present in promastigotes and amastigotes of *Leishmania amazonensis* and its surface expression increases during metacyclogenesis. Memórias Inst. Oswaldo Cruz.

[17] Berberich C., Machado G., Morales G., Carrillo G., Jimenez-Ruiz A., Alonso C. (1998). The expression of the *Leishmania infantum* KMP-11 protein is developmentally regulated and stage specific. Biochim. Biophys. Acta.

[18] Russo D.M., Turco S.J., Burns J.M., Reed S.G. (1992). Stimulation of human T lymphocytes by *Leishmania* lipophosphoglycan-associated proteins. J. Immunol..

[19] Jardim A., Funk V., Capriolit R.M., Olafson R.W. (1995). Isolation and structural characterization of the *Leishmania donovani* kinetoplastid membrane protein-11, a major immunoreactive membrane glycoprotein. Biochem. J..

[20] Carrillo E., Crusat M., Nieto J., Chicharro C., del Carmen Thomas M., Martínez E., Valladares B., Cañavate C., Requena J.M., López M.C. (2008). Immunogenicity of HSP-70, KMP-11 and PFR-2 leishmanial antigens in the experimental model of canine visceral leishmaniasis. Vaccine.

[21] Díaz-Regañón D., Roura X., Suárez M.L., León M., Sainz Á. (2020). Serological evaluation of selected vector-borne pathogens in owned dogs from northern Spain based on a multicenter study using a commercial test. Parasit. Vectors.

[22] Miró G., Checa R., Montoya A., Hernandez L., Dado D., Galvez R. (2012). Current situation of *Leishmania infantum* infection in shelter dogs in northern Spain. Parasit. Vectors.

[23] Solano-Gallego L., Cardoso L., Pennisi M.G., Petersen C., Bourdeau P., Oliva G., Miró G., Ferrer L., Baneth G. (2017). Diagnostic challenges in the era of canine *Leishmania infantum* vaccines. Trends Parasitol..

[24] Solano-Gallego L., Di Filippo L., Ordeix L., Planellas M., Roura X., Altet L., Martínez-Orellana P., Montserrat S. (2016). Early reduction of *Leishmania infantum*-specific antibodies and blood parasitemia during treatment in dogs with moderate or severe disease. Parasit. Vectors.

[25] Solano-Gallego L., Villanueva-Saz S., Carbonell M., Trotta M., Furlanello T., Natale A. (2014). Serological diagnosis of canine leishmaniosis: Comparison of three commercial ELISA tests (Leiscan^®^, ID Screen^®^ and Leishmania 96^®^), a rapid test (Speed Leish K^®^) and an in-house IFAT. Parasit. Vectors.

[26] Esteve L.O., Saz S.V., Hosein S., Solano-Gallego L. (2015). Histopathological findings and detection of toll-like receptor 2 in cutaneous lesions of canine leishmaniosis. Vet. Parasitol..

[27] Riera C., Valladares J.E., Gállego M., Aisa M.J., Castillejo S., Fisa R., Ribas N., Carrió J., Alberola J., Arboix M. (1999). Serological and parasitological follow-up in dogs experimentally infected with *Leishmania infantum* and treated with meglumine antimoniate. Vet. Parasitol..

[28] Cecílio P., Pérez-Cabezas B., Fernández L., Moreno J., Carrillo E., Requena J.M., Fichera E., Reed S.G., Coler R.N., Kamhawi S. (2017). Pre-clinical antigenicity studies of an innovative multivalent vaccine for human visceral leishmaniasis. PLoS Negl. Trop. Dis..

[29] Iborra S., Solana J.C., Requena J.M., Soto M. (2018). Vaccine candidates against *Leishmania* under current research. Expert Rev. Vaccines.

[30] Goldring J.P.D. (2019). Measuring protein concentration with bbsorbance, Lowry, Bradford coomassie blue, or the Smith bicinchoninic acid assay before electrophoresis. Methods Mol. Biol..

[31] Solano-Gallego L., Montserrrat-Sangrà S., Ordeix L., Martínez-Orellana P. (2016). *Leishmania infantum*-specific production of IFN-γ and IL-10 in stimulated blood from dogs with clinical leishmaniosis. Parasit. Vectors.

[32] Ramírez L., de Moura L.D., Mateus N.L.F., de Moraes M.H., do Nascimento L.F.M., de Jesus Melo N., Taketa L.B., Catecati T., Huete S.G., Penichet K. (2020). Improving the serodiagnosis of canine *Leishmania infantum* infection in geographical areas of Brazil with different disease prevalence. Parasite Epidemiol. Control.

[33] Freitas Y.B.N., Sousa C., Magalhaes J.M.E., Sousa M.L.R., D’Escoffier L.N., Valle T.Z., Goncalves T.C.M., Gil-Santana H.R., Kazimoto T.A., Amora S.S.A. (2018). Natural infection by *Trypanosoma cruzi* in triatomines and seropositivity for Chagas disease of dogs in rural areas of Rio Grande do Norte, Brazil. Rev. Soc. Bras. Med. Trop..

[34] Roque A.L., Xavier S.C., Gerhardt M., Silva M.F., Lima V.S., D’Andrea P.S., Jansen A.M. (2013). *Trypanosoma cruzi* among wild and domestic mammals in different areas of the Abaetetuba municipality (Pará State, Brazil), an endemic Chagas disease transmission area. Vet. Parasitol..

[35] Thomas M.C., Garcia-Perez J.L., Alonso C., Lopez M.C. (2000). Molecular characterization of KMP11 from *Trypanosoma cruzi:* A cytoskeleton-associated protein regulated at the translational level. DNA Cell Biol..

[36] Pinelli E., Killick-Kendrick R., Wagenaar J., Bernadina W., Del Real G., Ruitenberg J. (1994). Cellular and humoral immune responses in dogs experimentally and naturally infected with *Leishmania infantum*. Infect. Immun..

[37] Pinelli E., Van Der Kaaij S.Y., Slappendel R., Fragio C., Ruitenberg E.J., Bernadina W., Rutten V.P.M.G. (1999). Detection of canine cytokine gene expression by reverse transcription- polymerase chain reaction. Vet. Immunol. Immunopathol..

[38] Santos-Gomes G.M., Rosa R., Leandro C., Cortes S., Romão P., Silveira H. (2002). Cytokine expression during the outcome of canine experimental infection by *Leishmania infantum*. Vet. Immunol. Immunopathol..

[39] Do Nascimento P.R.P., Martins D.R.A., Monteiro G.R.G., Queiroz P.V., Freire-Neto F.P., Queiroz J.W., Morais Lima Á.L., Jeronimo S.M.B. (2013). Association of pro-inflammatory cytokines and Iron Regulatory Protein 2 (IRP2) with *Leishmania* burden in canine visceral leishmaniasis. PLoS ONE.

[40] de Almeida Leal G.G., Roatt B.M., de Oliveira Aguiar-Soares R.D., Carneiro C.M., Giunchetti R.C., Teixeira-Carvalho A., Martins-Filho O.A., Francisco A.F., Cardoso J.M., Mathias F.A.S. (2014). Immunological profile of resistance and susceptibility in naturally infected dogs by *Leishmania infantum*. Vet. Parasitol..

[41] Basu R., Bhaumik S., Basu J.M., Naskar K., De T., Roy S. (2005). Kinetoplastid, membrane protein-11 DNA vaccination induces complete protection against both pentavalent antimonial-sensitive and -resistant strains of *Leishmania donovani* that correlates with inducible nitric oxide synthase activity and IL-4 generation: Evidence for mixed Th1-and Th2-like responses in visceral leishmaniasis. J. Immunol..

[42] Carvalho L.P., Passos S., Dutra W.O., Soto M., Alonso C., Gollob K.J., Carvalho E.M., Ribeiro De Jesus A. (2005). Effect of LACK and KMP11 on IFN-γ production by peripheral blood mononuclear cells from cutaneous and mucosal leishmaniasis patients. Scand. J. Immunol..

[43] Singh O.P., Sundar S. (2014). Whole blood assay and visceral leishmaniasis: Challenges and promises. Immunobiology.

